# Laparoscopy with transverse-abdominal extra-fascial hysterectomy for early-stage endometrial carcinoma, obesity, and large uterus: A case report

**DOI:** 10.1097/MD.0000000000035981

**Published:** 2023-11-10

**Authors:** Jian Zou, Yang Li, Changkun Zhu

**Affiliations:** a Department of Gynecology and Oncology, Women’s Hospital, Zhejiang University School of Medicine, Hangzhou, Zhejiang Province, China; b Department of Zhejiang Provincial Key Laboratory of Precision Diagnosis and Therapy for Major Gynecological Diseases, Women’s Hospital, Zhejiang University School of Medicine, Hangzhou, Zhejiang Province, China.

**Keywords:** case report, endometrial carcinoma, laparoscopy combined with transverse-abdominal surgery, large uterus, obesity

## Abstract

**Rationale:**

Removal of a large uterus poses a challenge in minimally invasive surgery for patients with early-stage endometrial cancer. This manuscript presents 3 cases performed the improved surgical procedure with minimal trauma.

**Patient concerns:**

Three patients with obesity (Body Mass Index: 31.93, 30.06, and 51.82 kg/m^2^) and large uterus (7.3 × 8.0 × 7.6 cm, 8.5 × 8.9 × 8.5 cm, and 8.3 × 10.1 × 6.9 cm) visited our hospital because of vaginal bleeding, and received dilation and curettage. Pathological examination revealed endometrial carcinoma.

**Diagnoses:**

Endometrial carcinoma, obesity.

**Intervention:**

Laparoscopy and transverse-abdominal extra-fascial hysterectomy were performed. First, we performed bilateral adnexectomy, pelvic lymph node dissection, and para-aortic lymph node sampling, and exposed and separated the para-uterine tissue and bladder before cutting off the uterus from the vagina through laparoscopy. Second, we made a 10 cm suprapubic transverse incision in the lower abdomen, clamped the vagina using right-angle forceps to follow the principle of tumor-free technique, placed the uterus in a surgical bag for retrieval the uterus immediately from the incision.

**Outcome:**

All 3 patients underwent intestinal recovery for 24 hours post operation; 50 mL blood was lost during the operation with a well-healing wound and no complication. Till date, there has been no recurrence or metastasis in any of them.

**Lessons:**

Improving the surgical procedure could enhance safety and ease of operation even in cases of obesity and a large uterus.

## 1. Introduction

Approximately 70% of endometrial cancers are attributable to obesity.^[1]^ Laparoscopy, robotic extra-fascial hysterectomy, bilateral adnexectomy, and bilateral salpingectomy are recommended for early-stage endometrial carcinoma, with the advantages of good visual field exposure and less trauma and injury.^[2]^ However, transvaginal removal of the intact uterus is limited for patients with endometrial cancer, obesity, and a large uterus. Here, we present 3 cases of endometrial cancer with obesity and a large uterus treated laparoscopically combined with transverse-abdominal surgery.

## 2. Case report

### 2.1. Case 1

A 59-year-old, gravida 3 para 2 woman was admitted to our hospital with the complained of irregular vaginal bleeding for 2 years. Two years prior, the patient had experienced minimal spontaneous irregular vaginal bleeding that was not associated with dizziness or abdominal pain. Two weeks prior, she was diagnosed with complex atypical endometrial hyperplasia and cancer by complete endometrial curettage. She was additionally diagnosed with obesity (body mass index [BMI]: 31.93 kg/m2) and had hypertension for 3 years. Her brother had died of cholangiocarcinoma.

The patient’s vital signs were within the normal limits. Gynecological examination of the pelvis revealed an enlarged uterus with a regular outline and medium consistency, having preserved mobility and no pain on palpation. No mass or pain was observed upon palpation of the bilateral adnexa.

Her preoperative carbohydrate antigen 125 (CA125) level was 124.2 U/mL (< 25 U/mL), and carbohydrate antigen 19-9, alpha-fetoprotein (AFP), and carcinoembryonic antigen levels were within normal limits.

**Figure 1.**
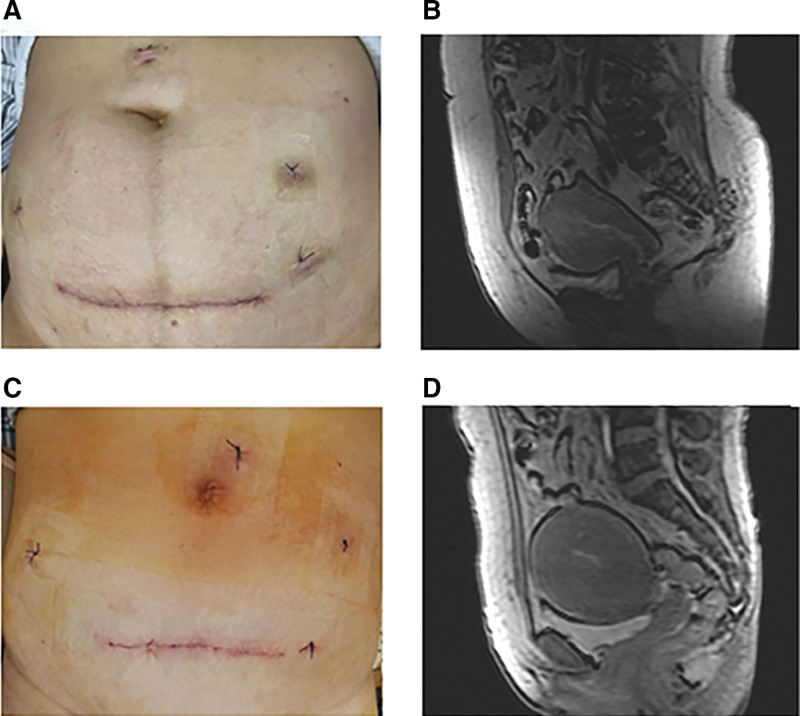
The incision and MRI image of case 1 (A and B) and case 2 (C and D) patients. (A) The incision of the lower abdomen of Case 1, (B) the size of her uterus was 7.3 × 8.0 × 7.6 cm and there was a 6.6*4.5*4.8 cm space-occupying shadow in uterine cavity myometrium, with unclear boundary, more than 1/2 the thickness of myometrium invasion in MRI image, (C) the incision of the lower abdomen of case 2, and (D) the size of her uterus was 8.5 × 8.9 × 8.5 cm and the tumor in uterine cavity infiltrate the superficial muscle layer in MRI image. MRI = magnetic resonance imaging.

Transvaginal ultrasound revealed the size of uterus to be 7.3 × 8.0 × 7.6 cm. Enhanced magnetic resonance imaging (MRI) of pelvic cavity showed a 6.6 × 4.5 × 4.8 cm space-occupying shadow in uterine cavity myometrium, with unclear boundary, more than 1/2 the thickness of myometrium invasion (Fig. 1B). Computed tomography (CT) revealed the pelvic and para-aortic lymph nodes to not be enlarged.

Based on the clinical manifestations and pathological results, the patient was diagnosed with endometrial carcinoma (FIGO stage IB).

Laparoscopic surgery combined with transverse-abdominal uterine malignant tumor surgery (extra-fascial hysterectomy, bilateral adnexectomy, bilateral pelvic lymphadenectomy, and para-aortic lymph node sampling) was performed.

The post-operative immunohistochemical results indicated endometrioid carcinoma with deep muscle infiltration. Immunohistochemical staining revealed PMS2 (+), MSH6 (+), MLH1 (+), MSH2 (+), PAX2 (−), ER (++), PR (+), and P53 (wild-type +).

Postoperatively, the patient received radiotherapy after excluding contraindications in other hospital.

Postoperatively, the CA125 level decreased to 89.4 U/mL (< 25 U/mL). After radiotherapy, the patient was followed up every 3 months, and no evidence of recurrence has been observed since 3 years now.

### 2.2. Case 2

A 45-year-old patient was admitted to our hospital with the complained of irregular vaginal bleeding for 1 month. A month before presentation, the patient experienced excessive menstrual flow and abnormal vaginal bleeding for 20 days. The patient was diagnosed to have (intrauterine) endometrioid adenocarcinoma (G2) with squamous differentiation and smooth muscle tissue infiltration by complete endometrial curettage. She had a normal vaginal delivery and 2 abortions. She had no history of surgery. She was obese (BMI: 30.06 kg/m2) and had diabetes since 1 and a half years. The patient denied any family history of malignant tumors.

The vital signs were within normal limits. Gynecological examination revealed an enlarged uterus with a regular outline and moderate consistency, preserved mobility, and no pain on palpation. No mass or pain was observed upon palpation of the bilateral adnexa.

Her preoperative CA125 level was 35.4 U/mL (normal range: < 35 U/mL), carbohydrate antigen 19-9, AFP, and carcinoembryonic antigen levels were within normal limits.

Transvaginal ultrasound showed the size of uterus to be 8.5 × 8.9 × 8.5 cm. Enhanced MRI of the pelvic cavity showed that the tumor in the uterine cavity had infiltrated the superficial muscle layer (Fig. 1D). Abdominal CT revealed no enlargement of the pelvic or para-aortic lymph nodes.

Based on the clinical manifestations and pathological results, the patient was diagnosed with endometrial carcinoma (FIGO IA).

Laparoscopic surgery combined with transverse-abdominal uterine malignant tumor surgery (extra-fascial hysterectomy, bilateral adnexectomy, sentinel lymph node imaging labeling, sentinel lymph node dissection, and para-aortic lymph node sampling) was performed.

Post-operative immunohistochemical results indicated endometrioid carcinoma with superficial muscular infiltration. Immunohistochemical staining revealed the expression of PMS2 (+), MSH6 (+), MLH1 (+), MSH2 (+), PAX2 (+), ER (+++), PR (+++), and P53 (wild-type +).

Postoperatively, the patient had no need to undergo radiotherapy or chemotherapy.

Postoperatively, the CA125 level decreased to 26 U/mL (normal range: < 35 U/mL). The patient was followed up every 3 months for 1 to 3 years after surgery and then every 6 months for 3 to 5 years after surgery. No evidence of recurrence has been observed since 5 years now.

### 2.3. Case 3

A 35-old-patient was admitted to our hospital with the complained of irregular vaginal bleeding for more than 1 year. More than a year ago, the patient experienced irregular vaginal bleeding every day, and 2 months prior, the amount of bleeding had increased. One month ago, she had been diagnosed to have complex atypical endometrial hyperplasia with cancer based on hysteroscopy and complete endometrial curettage. She was unmarried and childless, and had no desire to conceive. The patient had no history of surgery. She was obese (BMI: 51.82 kg/m2). The patient denied any family history of malignant tumors.

The patient’s vital signs were within normal limits. Gynecological examination revealed an enlarged uterus. Her CA125 level before surgery was 100 U/mL (normal range: < 47 U/mL), carbohydrate antigen 19-9 was 66.3 U/mL (normal range: < 30 U/mL), human epididymal protein 4 was 161 pmol/L (normal range: 29.25–68.5 pmol/L), and AFP and carcinoembryonic antigen levels were within normal limits.

Transvaginal ultrasound showed the size of uterus to be 8.3 × 10.1 × 6.9 cm. Enhanced MRI of the pelvic cavity in another hospital revealed that the tumor in the uterine cavity had infiltrated the deep muscle layer. Abdominal CT showed that the lymph nodes were not enlarged.

Based on the clinical manifestations and pathological results, the patient was diagnosed with endometrial carcinoma (FIGO stage IB).

**Figure 2.**
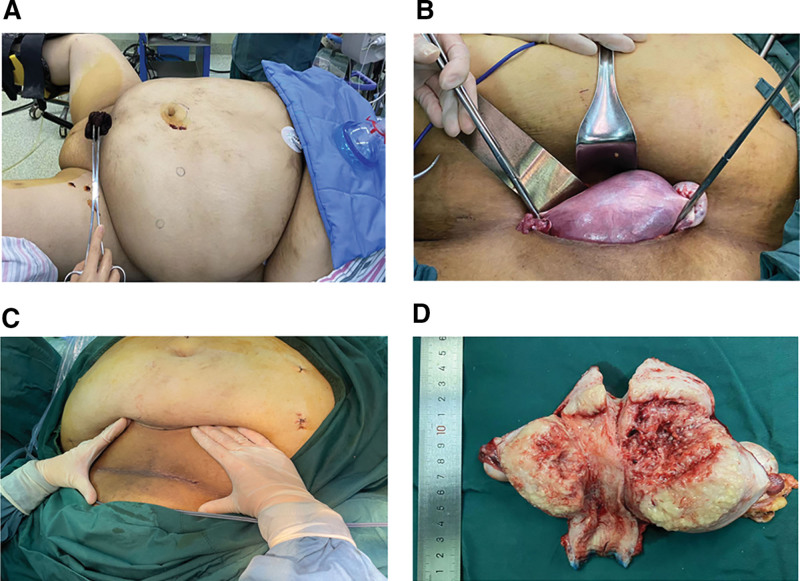
Intraoperative photograph of case 3 patient. (A) The abdomen of the patient, (B) the vagina was clamped using right-angled forceps to follow the principle of the tumor-free technique, and the uterus was removed immediately from the suprapubic transverse incision, (C) the suprapubic transverse incision, and (D) gross findings of the resected specimen: tumor in the uterine cavity.

Laparoscopic surgery combined with transverse-abdominal uterine malignant tumor surgery (extra-fascial hysterectomy, bilateral adnexectomy, pelvic lymph node dissection, and para-aortic lymph node sampling) was performed. The tumor in the uterine cavity was showed in Figure 2D.

The post-operative immunohistochemical results suggested endometrioid carcinoma with deep muscular infiltration. Immunohistochemical staining revealed expression of PMS2 (+), MSH6 (+), MLH1 (+), MSH2 (+), PAX2 (+), ER (++), PR (++), and P53 (wild-type +).

The uterus postoperatively, the patient received radiotherapy after excluding contraindications. The tumor marker (CA125) level was normal at 3 months post operation. The patient was followed up for half a year and no evidence of recurrence has been observed yet.

## 3. Discussion

Endometrial cancer is one of the most common malignant tumors in women, with an estimated 320,000 annual incidences_._^[3,4]^ Approximately 70% of the endometrial cancers are attributable to obesity. BMI is a measure of obesity (defined as BMI > 30 kg/m^2^ and < 35 kg/m^2^). Obesity is associated with a 2.6-fold increase in endometrial cancer risk, whereas severe obesity (BMI > 35 kg/m^2^) is associated with a 4.7-fold increase compared to that of normal weight (BMI < 25 kg/m^2^).^[5]^ It is still a challenge how to deal with the endometrial cancer patients with obesity, especially in those with a large uterus.

Minimally invasive surgery (MIS) is the standard surgical approach for comprehensive surgical staging of women with early-stage endometrial cancer.^[6]^ However, the transvaginal removal of an intact uterus is limited in those patients with a large uterus. Removal of a large uterus poses a challenge in MIS.^[7]^ Nowadays, total abdominal hysterectomy in patients is mainly performed in those endometrial carcinoma patients with large uterus and obesity, while may be challenging because of limited visibility and wound complications. Robot-assisted laparoscopy surgery is more commonly applied in those patients,^[8–10]^ with the obvious advantages of good visual field exposure, minimal invasion, and rapid recovery.^[11–13]^ Akiyo Kakibuchi had reported robot-assisted laparoscopic hysterectomy for early-stage endometrial cancer with massive uterine leiomyomas and significantly fewer intra- and post-operative complications.^[14]^ In their study, the umbilical trocar incision was extended by a 7 cm vertical incision on both sides, and the uterus was removed through the incision. However, in a retrospective review of 1027 patients (461 underwent laparoscopy and 566 underwent robot-assisted MIS), robot-assisted MIS was associated with poorer long-term patient outcomes.^[15]^

To understand the kind of incision that would be better for removing an intact uterus. A systematic review and meta-analysis demonstrated that vertical incisions were associated with a relative risk of 2.07 (95% CI 1.61–2.67) for wound complications compared to transverse incisions.^[16]^ And another study demonstrated that high transverse skin incisions may reduce the risk of wound complications in parturients with obesity.^[17]^ The abdominal wall of case 3 was very thick (Fig. 2A). The suprapubic transverse incision (Fig. 2C) additionally has the advantages of easier exposure and closure of the vagina using an angled clamp to follow the principles of tumor-free technique and placed the uterus in a surgical bag for retrieval the uterus immediately from the incision. Therefore, a suprapubic transverse incision may be more suitable than a vertical 1 since the former is closer to the vagina with better exposure of surgical field of vision.

Therefore, we performed laparoscopic surgery combined with transverse-abdominal surgery, since it is easier to expose and separate the para-uterine tissue and bladder laparoscopically. First, we performed bilateral adnexectomy, pelvic lymph node dissection, and para-aortic lymph node sampling through laparoscopy, exposing and separating the para-uterine tissue and bladder before cutting off the uterus from the vagina. Second, we made a 10 cm suprapubic transverse incision in the lower abdomen (Figs. 1A, C and 2C), clamped the vagina using right-angle forceps to follow the principle of tumor-free technique (Fig. 2B), and then placed the uterus in a surgical bag for retrieval the uterus immediately from the incision.

The average bleeding volume of the 3 patients was 50 mL, and the hemoglobin level decreased to < 4 to 5 g/L. The patients recovered well after the operation. None of the patients experienced any complication (Table 1). The incisions healed well, without fat liquefaction. One of the patients was followed up for 5 years, 1 for 3 years, and another for half a year; CA125 of all of them was normal, and there has been no sign of recurrence by B ultrasound and CT scan till date.

**Table 1:** All three patients had rapid recovery with no post-operative complication.

Cases	Intestinal recovery	Antibiotic use	Pre-operation Hb (g/L)	Post operation Hb (g/L)	Blood loss (mL)	Complications
Case 1	24 h	1 d	129	130	50	None
Case 2	24 h	1 d	98	94	50	None
Case 3	24 h	5 d	91	86	50	None

HB = hemoglobin.

For patients with endometrial carcinoma along with obesity and a large uterus, compare to total abdominal hysterectomy and robot-assisted laparoscopy surgery, the improved surgery has the advantages of easy manipulation, no need for special equipment, being economic, faster healing of transverse incision than vertical incision, faster post-operative recovery, better wound healing, shorter hospitalization time, better outcome, and especially removing the intact uterus from the abdominal incision following the principle of tumor-free technique, thereby improving prognosis and reducing recurrence.

Our study had some limitations. Since we only presented 3 cases, an increase in the number of cases and longer follow-up periods are needed to determine the most suitable surgical approach, considering factors such as smaller wounds, shorter operative times, and better outcomes.

## 4. Conclusion

Laparoscopic surgery combined with transverse-abdominal surgery is suitable for patients with early-stage endometrial cancer, obesity, and a large uterus.

## Acknowledgments

The authors would like to thank the patients and their families for their participation and their dedication in providing follow-up information. We also thank the entire study group for regularly updating the registry. A patient informed consent was acquired.

## Author contributions

**Conceptualization:** Changkun Zhu.

**Data curation:** Jian Zou.

**Investigation:** Yang Li.

**Resources:** Yang Li.

**Supervision:** Changkun Zhu.

**Writing – original draft:** Jian Zou.

**Writing – review & editing:** Jian Zou, Yang Li.
